# Supplementary material for: Relationship between students’ attitude towards, and performance in mathematics word problems
Source: PLoS One. 2024 Feb 6;19(2):e0278593. doi: 10.1371/journal.pone.0278593 (PMC10846736; doi:10.1371/journal.pone.0278593)
Supplement: Attachment — Submitted filename: review comments.edited.docx [file pone.0278593.s002.docx]

This research seems to be interesting, but the author did not introduce it following rigorous academic norms. The theoretical basis, methodology, analysis and discussion of the results have not been fully delivered. In the literature review section, many ideas are vague and need to be expanded. In the methodology section, does the research design effectively respond to the purpose of the research, and does each subsection follow a logical sequence? The discussion section lacks systematic analysis, as well as critical discussion. In the Results section, there is no response to the purpose of the study. The following are some specific comments including textual and logical issues:

P3, 1.1 Verschaffel et al., 2010),

P5, 1.3 there is no “in the middle of the sentence.

P2 and P3, there are many references supporting one statement, which is a very general conclusion, for example, "some empirical findings report students’ attitudes towards specific units or topics in mathematics to enhance the learning of that specific content and mathematics”, and “Yet, some empirical studies have shown existence of significant

relationships between students’ attitude and performance in mathematics”. Do they say the same thing or have the same effect? And could you kindly clarify this statement and make those references indicating the specific results?

P3, “it appears that multiple factors ranging from students’ demographic factors to teachers’ classroom instructional practices influence students’ attitude towards learning mathematics" What multiple factors? I would suggest authors indicate this clearly.

P4, “Despite numerous difficulties encountered by students in algebraic inequalities (e.g., Fernández & Molina, 2017; Molina et al., 2017;Bazzini & Tsamir, 2004; Tsamir & Almog, 2001; Tsamir & Bazzini, 2004, 2006; Tsamir & Tirosh, 2006), a combination of methods (strategies) rather than one specific method can be applied to overcome specific students’ learning challenges.” These sentences seem not to be connected with the prior sentences. Please re-organize this part so that it could be aligned with this section.

Section 1.2 is too simple, authors should expand some points, for example, “Previous empirical studies have revealed that LP and/or related concepts are not only difficult for learners but also challenging to teach (Awofala, 2014; Goulet-Lyle et al., 2020; Kenney et al., 2020; Verschaffel et al., 2020a, 2020b).” What difficulties did the students encounter, cognitive or non-cognitive? “Different factors account for learners’ challenges in mathematics word problems?” What are these different factors? Is there existing research on attitudes towards Mathematics Word Problems? What kind of attitudes students held?

P6, not sure how these two theoretical frameworks connected. Could you clarify how "expectancy-value model theory within the constructivism paradigm” works?

P7, “Active Learning Heuristic Problem Solving Strategies" is here for the first time. More information should be provided in the literature review about these strategies.

Methodology, authors should make sure the correct methodology, “A quantitative survey research design” or “A quasi-experimental pre-test, post-test, non-equivalent, non-randomized control group study design". Please check the methodology and method. They are different.

P9, Treatment group vs. Experimental group, please use the same term throughout the context.

P9, “The learning in the comparison group was purely conventional, and teachers did not follow proper and organized strategies as was the case with the treatment group.” It seems that the treatment group has been involved in a more scientific, systematic, and better teaching method than the comparison group, so it is certain that the treatment group will achieve better results. In this case, the conclusion is obvious.

“Empirical studies (e.g., Abdul et al., 2010; Sari et al., 2012) have found that the stated materials enhance students’ conceptual understanding and critical thinking abilities. These strategies were further integrated in problem solving strategies (Polya, 2014) by ensuring that students understand the LP word problem, devise a plan, adequately carry out the plan and finally look back to verify solution sketches (see Appendix 3 for PS01, PS02, PS03, PS04). To ensure that students minimize errors and misconceptions, the learning of LP was further integrated with Newman Error Analysis (NEA) model (Mushlihah, 2018)." Those statements should be put in the literature review.

“The instrument was designed by the researcher and validated by experts in mathematics education as an error analysis tool to provide teachers with a framework to consider the underlying reasons why students answer mathematics word problems incorrectly. The teachers emphasized question reading and decoding, comprehension, transformation, process skills and encoding (see Appendix 3 for NEA01, NEA02, NEA03, NEA04 and NEA05).” You started to say new points. This paragraph contains two many ideas: strategies, instruments, coding.

P10. “the post attitude questionnaire and achievement test were administered to both groups, and analysis was done by comparing and contrasting students’ feedback on attitudinal constructs and the post-test scores." Not sure what the achievement test is. You should clearly state how many instruments you apply and how many hours implement the treatment group. Who implemented it in the treatment group and comparison group?

For table 4 and table 5, there is not enough explanation, what statistical method is used, and how to explain the data?

Again, the description of table8,9,10 is also very simple and unclear.

The main problem in the discussion part is that there is a lack of comparison with previous literature, and critical discussion, as with the total number of literature, the author needs to make a more detailed comparison with the relevant literature instead of citing a lot of literature.

The conclusion section does not respond well to the purpose of the study.
